# The health benefits of a targeted cash transfer: The UK Winter Fuel Payment

**DOI:** 10.1002/hec.3666

**Published:** 2018-05-09

**Authors:** Thomas F. Crossley, Federico Zilio

**Affiliations:** ^1^ Department of Economics University of Essex, Colchester, UK, and Institute for Fiscal Studies London UK; ^2^ Faculty of Economics University of Cambridge Cambridge UK

**Keywords:** benefits, biomarkers, health, heating, regression discontinuity

## Abstract

Each year, the UK records 25,000 or more excess winter deaths, primarily among the elderly. A key policy response is the “Winter Fuel Payment” (WFP), a labelled but unconditional cash transfer to households with a member above the female state pension age. The WFP has been shown to raise fuel spending among eligible households. We examine the causal effect of the WFP on health outcomes, including self‐reports of chest infection, measured hypertension, and biomarkers of infection and inflammation. We find a robust, 6 percentage point reduction in the incidence of high levels of serum fibrinogen. Reductions in other disease markers point to health benefits, but the estimated effects are less robust.

## 1 INTRODUCTION

Each year, the UK experiences excess winter mortality (EWM). In 2014/2015, the number of excess winter deaths in England and Wales was estimated at 43,900, the highest level since 1999. EWM has also been documented in Europe (Eng & Mercer, 1998; Keatinge & Donaldson, 1995; Kunst, Looman, & Mackenbach, 1993; Mackenbach, Kunst, & Looman, 1992; Rose, 1966), the USA (Kloner, Poole, & Perritt, 1999; Lanska & Hoffmann, 1999), and Asia (Chen, 1993; Ornato, Siegel, Craren, & Nelson, 1990). Most EWM occurs among the elderly and is due to respiratory and circulatory diseases (Donaldson, 2010; Lloyd, 2013; ONS Statistical Bulletin 2014/2015). 1In the winter of 2014/2015, 36% of EWM was attributable to respiratory disease and 22.5% to cardiovascular disease (ONS Statistical Bulletin 2014/2015). In addition to EWM, cold weather is associated with increased demands on health care systems through increased incidence of illness requiring hospitalisation or other treatment.

Although outdoor temperatures may play a role in EWM, 2The strong association between exposure to outdoor cold temperatures and mortality or morbidity is well documented in the epidemiological literature (Curwen, 1997; Keatinge, 1986, 2002; Wilkinson et al., 2001). living in a cold indoor environment has a direct impact on the health of elderly people (Dear & McMichael, 2011; Marmot, Geddes, Bloomer, Allen, & Goldblatt, 2011; Rudge & Gilchrist, 2007; Wilkinson, Armstrong, & Landon, 2001). 3Wilkinson et al. (2001) show that deaths attributable to cardiovascular diseases are 23% higher in winter than the rest of the year and give evidence of a positive association of the EWM with the age of the property and the thermal inefficiency of the buildings. Rudge and Gilchrist (2007) find that their fuel poverty index that includes an energy efficiency rates and income is a strong predictor of the excess winter morbidity measured with number of emergency respiratory hospital admissions. In the UK, a key policy response to EWM is the Winter Fuel Payment (WFP). The WFP is a labelled but unconditional cash transfer to households containing an older person (male or female) above the female state pension age. The stated intent of the policy is to help the elderly deal with the cost of keeping their dwelling warm (Kennedy & Parkin, 2016; Lloyd, 2013), and the labelling of this cash transfer has been shown to be effective in inducing eligible households to increase fuel spending (Beatty, Blow, Crossley, & O'Dea, 2014). 4Beatty et al. (2014) estimate that eligible households spend 47% of their WFP on fuel. If the payment was treated as other income, eligible household would be expected to spend 3% of the payment on fuel. However, the key policy question is whether the WFP improves health of elderly people living in eligible households, and there is little evidence on this point.

The female state pension age, and hence the age cut‐off for WFP eligibility, was 60 prior to 2010. It then began to increase so that it will equal the male state pension age of 65 in 2018, and then both will rise to 67 by 2028. Most EWM occurs among individuals over 75, leading Lloyd (2013) to propose that increases in the eligibility age could reduce the financial cost of the WFP with minimal if any reduction in health benefits. But there has been no direct evidence on the health benefits foregone through recent increases in the age cut‐off or health benefits that may be lost through further increases.

This paper reports the first tests for health benefits of the WFP based on individual‐level data. We measure health outcomes in the Health Surveys for England (HSE), the Scottish Health Survey (SHeS), and the English Longitudinal Study on Ageing (ELSA). These studies include nurse visits, allowing us study biomarkers and physical measures as well as self‐reports. To estimate the causal effect of the WFP, we follow Beatty et al. (2014) in employing a regression discontinuity design (RDD). The RDD is thought to be the most convincing of quasi‐experimental designs (Lee & Lemieux, 2010). 5The RDD was first introduced in the education literature (see Thistlethwaite & Campbell, 1960) and has been widely adopted in economics (Lee & Lemieux, 2010) An RDD is possible where there is cut‐off in eligibility for treatment, as there is for the WFP: In the period we study, households with no member aged 60 or above are ineligible. 6Take‐up of the WFP is very high, so that there is little difference between eligibility and receipt (see Beatty et al. (2014) for further discussion). An RDD estimates the causal effect of treatment by comparing outcomes just below and above the eligibility cut‐off. It estimates a local average treatment effect—at the eligibility cut‐off. Thus, our design estimates the causal effect of the WFP on the health of individuals living in households where the oldest member of the household is 61. This is precisely the age group that lost any health benefits of the WFP as the eligibility age was incrementally increased from 2010, so our empirical strategy directly answers a key policy question.

To the best of our knowledge, Iparraguirre (2014) is the only prior assessment of the health benefits of the WFP. Using aggregate mortality data, Iparraguirre documents a decline in EWM in 2000/2001, coincident with the introduction of the universal WFP. 7The WFP was introduced in 1997, but it was initially means‐tested and the payment significantly smaller. In 2000/2001, it took its current form (a universal payment to all households containing a person above the female state pension age of between 200 and 300 pounds). EWM fluctuates significantly from year‐to‐year with changing winter weather conditions and viral environment. Using time‐series econometric techniques, Iparraguirre finds a structural break in the EWM time series for England and Wales in 2000/2001 and estimates that half of the reduction in the EWM in that year can be attributed to the introduction of the WFP. This is an important finding, but it does rest on the ability of the econometric methods to distinguish the policy effects from the very substantial year‐on‐year fluctuations in EWM. Moreover, the aggregate EWM time series is necessarily silent on health effects that may precede mortality, and on benefits to particular groups. We add to the evidence base significantly by using a convincing quasi‐experimental design in conjunction with individual level data; by considering a variety of measures of circulatory and respiratory illness, including biomarkers 8Biomarkers have been increasingly drawing attention in the economic literature as an objective measure of health and a complement of self‐reported health measures (see Evans & Garthwaite, 2014; Jürges, Kruk, & Reinhold, 2013; Michaud, Crimmins, & Hurd, 2016); and by testing for health benefits particularly among the group that have been made ineligible by recent changes to the age cut‐off.

We estimate the effect of the WFP on circulatory and respiratory illness measured four ways: self‐reports of chest infection, nurse‐measured hypertension, and two blood biomarkers of infection and inflammation. Our principal finding is that, among those living in a household that just qualifies for the payment, the WFP leads to a 6 percentage point reduction in the incidence of high levels of serum fibrinogen (on a base of 12%). High levels of fibrinogen are considered to be a marker of current infection and are also associated with chronic pulmonary disease. This effect is statistical significant (*p* < .01) and very robust. For the other health measures we consider, although point estimates suggest health benefits, the estimated effects are less robust to changes in sample or specification, and rarely statistically significant at conventional levels.

In the next section, we provide further detail on our data, outcomes measures, identification strategy, and methods. Section 3 presents our results. Section 4 contains additional discussion of the findings.

## 2 DATA AND METHODS

### 2.1 Data

The analysis reported in this paper is based on data from the HSE (2001, 2003, 2004, 2005, 2006, and 2009; see NatCen, 2010a, 2010b, 2010c, 2011, 2015), 9HSE 2001 contains hypertension measure and self‐reports of chest infection only (not the biomarkers). the SHeS (2003, 2008, and 2009; see JHSU, 2016 and ScotCen, 2016a, 2016b), and the ELSA (Wave 2, 2004–2005, and Wave 4, 2008–2009; see Marmot et al., 2017). The HSE and the SHeS are annual cross‐sectional surveys of the health conditions of the population in England and Scotland. ELSA is a longitudinal survey that captures the population in England aged 50 and over. The ELSA sample is derived from the 1998, 1999, and 2001 HSE. In all three surveys, a face‐to‐face interview is followed by a nurse visit. In the interview, the respondents answer questions on their general health, smoking status and alcohol consumption, and other individual characteristics such as education and employment status. After the interview, an appointment for the nurse visit is arranged. In the visit, a trained nurse asks questions on the health condition of the respondent, takes blood and saliva samples, and reads the blood pressure and several other measures (height, weight, waist, hip, lung function, and grip strength). Blood samples are sent to an external laboratory for analysis. ELSA, HSE, and SHeS data contain several biomarkers that are recovered from the analysis of the blood samples. Among the biomarkers reported, there are two that are useful for our analysis because they are correlated with inflammation processes and infection and are markers of circulatory and respiratory illness. These are C‐reactive protein (CRP) and fibrinogen.

Over the period 2003–2009, the female state pension age was 60, 10In the same period, the male state pension age was 65. and so any household with a member over age 60 qualified for the WFP. Following Beatty et al. (2014), we restrict the sample to single men and couples in which the man is the oldest in the household. We discard single women and couples in which the woman is the oldest member of the household because such households qualify for the WFP and the woman's state pension simultaneously. In household in which the oldest member is male, eligibility for the WFP and the first state pension are not coincident. Thus, we avoid any confounding effect of receipt of the state pension on the fuel expenditure and the health outcomes of the elderly. We have not identified any other potential confounding policy directed at persons aged 60 and above. For example, free flu vaccinations are offered to elderly aged 65 or over (Department of Health, 2000).

### 2.2 Health outcomes

We study four measures of circulatory and respiratory illness:

- self‐reports of chest infection in the last 3 weeks;
- hypertension;
- serum values of CRP in excess of 10;
- serum values of fibrinogen equal or in excess of 4.

During the nurse visit, respondents are asked whether they have experienced any respiratory infection in the preceding 3 weeks (influenza, pneumonia, bronchitis, or a severe cold). This self‐reported outcome is available only in ELSA and SHeS.

Our second outcome is hypertension, a risk factor for strokes and heart attacks. The World Health Organization defines hypertension as systolic blood pressure of 140 mm Hg or above and/or diastolic blood pressure of 90 mm Hg or above (World Health Organization, 2013). Repeated exposure to a cold environment results in an increase in the blood pressure, and high values of systolic and/or diastolic blood pressure (hypertension) is a predictor of heart disease and stroke (Collins, Easton, Belfield‐Smith, Exton‐Smith, & Pluck, 1985; Collins et al., 1990; Fraser, 1986; Hofman, Feinleib, Garrison, & van Laar, 1983; Wilson et al., 1998). In our sample, around the cut‐off age for WFP eligibility, about 35% of respondents are hypertensive.

The other outcomes in our study are CRP and fibrinogen, two acute‐phase biomarkers. Serum concentrations of these two biomarkers increase sharply during an inflammatory process. CRP is a blood plasma protein that is indicative of inflammation and infection and a risk predictor of cardiovascular disease. It is considered an indicator of bacterial infection, pneumonia, and tissue damage (Pepys & Hirschfield, 2003; Pearson et al., 2003; Simon, Gauvin, Amre, Saint‐Louis, & Lacroix, 2004; Tillett & Francis, 1930). The median of CRP in our sample is 1.7 mg/L, but its distribution is highly skewed. The value rises within few hours of disease onset. Inflammation and bacterial infection can produce a rise in CRP values up to 1,000‐fold (Gruys, Toussaint, Niewold, & Koopmans, 2005; Pepys & Hirschfield, 2003). Fibrinogen is a coagulation protein produced by the liver that helps the body in the formation of blood clots. The normal range of fibrinogen is 2–4 g/L, but the concentration increases up to threefold in the presence of an inflammatory process, infection, or tissue damage (Fenger‐Eriksen, Lindberg‐Larsen, Christensen, Ingerslev, & Sørensen, 2008; Gruys et al., 2005; Schmaier, 2012). High concentrations of fibrinogen are also strongly associated with chronic obstructive pulmonary diseases and moderately with coronary heart diseases (Danesh et al., 2005; Duvoix et al., 2013; Mannino et al., 2015). In the epidemiological literature, a value of the CRP in excess of 10 is taken as evidence that a person has an active infection or inflammatory process. Because high values of CRP or fibrinogen are considered evidence of current infection, Epidemiologists often discard observations with these high values in order to focus on chronic processes (Pearson et al., 2003).

However, as our interest is whether the WFP plays a role in reducing the incidence of a respiratory or circulatory disease among the elderly, extreme values (in excess of 10 for CRP) are the appropriate object of our analysis. The epidemiological literature has not defined an equivalent disease threshold for fibrinogen, but we take values in excess of the top the standard range (2–4 g/L) as evidence of current infection or inflammation.

### 2.3 Regression discontinuity design

The RDD allows health outcomes to vary with the “forcing variable,” which is the age of the oldest person in the household of subject *i* at the time of the interview *t*. Denote this by (*A*_*it*_). The econometric model includes smooth functions of the value of the forcing variable relative to the cut‐off age (*A*_*it*_ *−60*). It also includes an indicator (or dummy) variable, *D*_*it*_, for whether a respondent's household was eligible for the last WFP payment before the interview at which health outcomes were measured. Finally, it includes additional covariates *X*_*it*_, to increase the precision of the estimator by capturing individual background variation in health (unrelated to the WFP). 11The assumption behind the RDD is that all health determinants apart from WFP eligibility should evolve smoothly with *A*_*it*_, including (but not limited to) the covariates *X*_*it*_. For the covariates *X*_*i*_, we can test this by estimating an RDD in *X*_*i*_. There should be no discontinuity in the *X*_*i*_ at the 60 or, equivalently, observables should be balanced between eligible and not eligible in the region of the cut‐off (analogous to covariate balance in a randomised trial). We have tested this and cannot reject for balance for any of the included covariates *X*_*i*_. This implies that the point estimate of the treatment effect is not affected by the inclusion of the covariates, which we confirm in our robustness checks. The covariates can, however, improve precision by reducing the unexplained variation in the outcome variable (again, as in a randomised trial). Our covariates include type of households (single or couple), individual characteristics (gender, smoker status, alcohol consumption, education, income, and employment status), anthropometric measurements (body mass index and waist circumference), dummies for each wave (year) of each survey, and month of nurse visit.

Thus, the econometric model is

$$H_{\mathrm{it}}=\beta_{0}+f\left(A_{\mathrm{it}}-60\right)+\tau D_{\mathrm{it}}+f\left(A_{\mathrm{it}}-60\right)\times D_{\mathrm{it}}+X_{\mathrm{it}}\gamma +e_{\mathrm{it}}.$$

We employ both linear and quadratic functions for *f*(). The model is estimated by ordinary least squares. Note that all of the health measures (*H*_*it*_) we consider are binary so that *E*[*H*_*it*_] = *Prob*(*H*_*it*_ = 1),   and this is a linear probability model. The parameter of interest is τ, which measures the local causal effect of the WFP on *Prob*(*H*_*it*_ = 1), around the cut‐off. Formally,

$$\tau =\lim_{A↓60}E\left[H_{\mathrm{it}}|A_{\mathrm{it}}=60,X_{\mathrm{it}}=x\right]-\lim_{A↑60}E\left[H_{\mathrm{it}}|A_{\mathrm{it}}=60,X_{\mathrm{it}}=x\right].$$

As the *H*_*it*_ are measures of illness, if the WFP improves health, τ should be negative. We report standard errors that are robust to heteroscedasticity and clustering by the age in years of the oldest member of the respondent's household. Because we look at multiple health outcomes, we also report *p*‐values adjustment for multiple testing using the Romano–Wolf algorithm (Romano & Wolf, 2016).

The WFP was between £200 and £300 (about 300–450 U.S. dollars) during the period 2002–2009 12From 2000 to 2007, the WFP was £200 for most eligible households but £300 for households with an over‐80s member. In 2008, the WFP was temporarily uplifted to £250 for over 60s and to £400 for over 80s, but this increase was reversed in the budget of March 2011. The sharp increase in the payment at age 80 might seem to offer an opportunity for a second RDD, but our data are too sparse to the right of this cut‐off to implement this. when our data were collected, and it was paid in November–December. 13Recipients receive a letter at the time of the payment indicating that the cash transfer is to help with the cost of heating their home. Eligibility is determined by the age of the old household member in the preceding September. In our data, ages are recorded in years. That means, the WFP status of some households with the oldest member aged 60 or 61 can only be determined probabilistically (for details see the Supporting Information).

## 3 RESULTS

**Figure 1:**
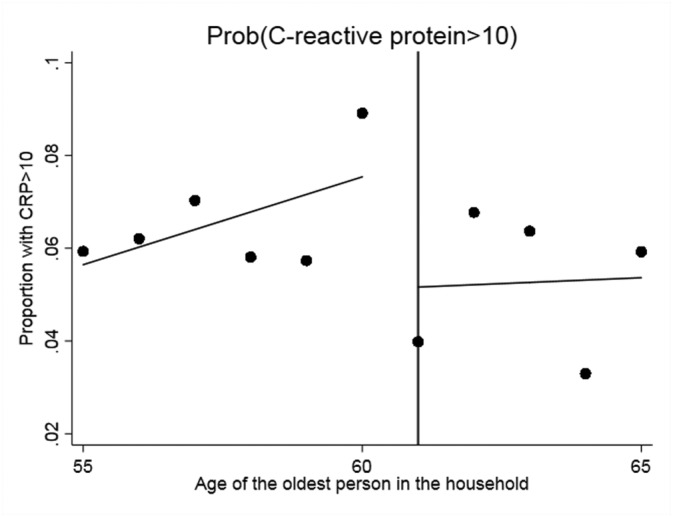
Effect of the Winter Fuel Payment on the probability of having a C‐reactive protein level larger than 10. Plotted points represent the incidence of (C‐reactive protein > 10) by year of age of the oldest person in the subject's household, with best fit regression lines to the left and right of the eligibility cut‐off. CRP = C‐reactive protein

**Figure 2:**
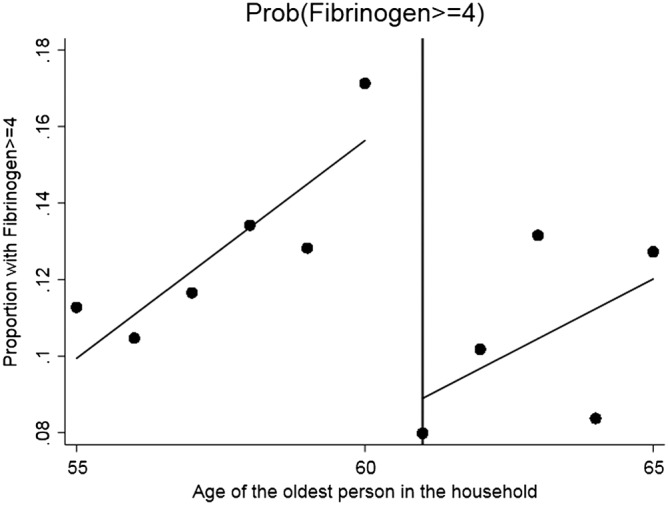
Effect of the Winter Fuel Payment on the probability of having a fibrinogen level equal or larger than four. Plotted points represent the incidence of (Fibrinogen ≥ 4) by year of age of the oldest person in the subject's household, with best fit regression lines to the left and right of the eligibility cut‐off

**Figure 3:**
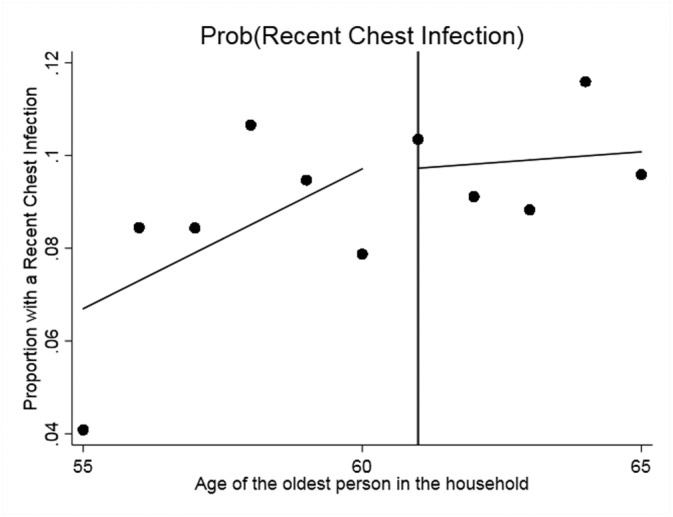
Effect of the Winter Fuel Payment on the probability of having had a recent chest infection. Plotted points represent the incidence of (self‐reported chest infection in the last 3 weeks) by year of age of the oldest person in the subject's household, with best fit regression lines to the left and right of the eligibility cut‐off

**Figure 4:**
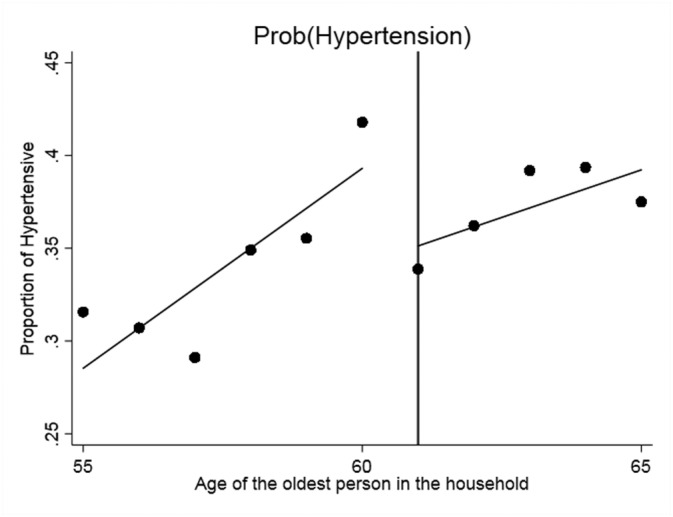
Effect of the Winter Fuel Payment on the probability of having hypertension. Plotted points represent the incidence of (hypertension) by year of age of the oldest person in the subject's household, with best fit regression lines to the left and right of the eligibility cut‐off

We begin with the standard graphical presentation of the RDD in Figures 1, 2, 3, 4. Each figure corresponds to one of our four measures of circulatory and respiratory illness. The vertical axis measures the incidence of illness. The horizontal axis measures the age of the oldest member of a respondent's household. Each plotted point is the average value of the illness measure for a given age of oldest household member (in years). As our illness measures are binary, this mean is an incidence (or empirical probability). The cut‐off for WFP eligibility (at age 61) is indicated in Figures 1, 2, 3, 4 by the vertical line, and separate least‐squares best‐fit lines are plotted to the left and the right of the cut‐off. A treatment effect is indicated by a discontinuity between these two best‐fit lines at the cut‐off.

Three of the four figures indicate decline in illness incidence with WFP eligibility. The exception is self‐reported chest infection. The plots also indicate that the paths of illness incidence across age of the old household member are quite noisy, with significant year on year fluctuations. Although we have substantial sample sizes (each point represents about 400 observations), we are modelling relatively rare events (in our sample 5.5% of the elderly just below the cut‐off present a CRP value larger than 10% and 12.5% a fibrinogen value in excess of four, see Table 1). To assess the magnitudes and statistical significance of the discontinuities visible in these figures, we turn to formal RDD estimates (as described above). These are reported in Table 2.

**Table 1:** Descriptive statistics

	Age window	Median	90th percentile	Prob(illness)
C‐reactive protein	58–63	1.7	7	Prob(CRP > 10) 0.055
Fibrinogen	58–63	3.1	4	Prob(Fib ≥ 4) 0.125
Hypertension	58–63	—	—	Prob(hypertension) 0.353
Self‐reported chest infection	58–63	—	—	Prob(chest infection) 0.101

*Note*. C‐reactive protein, fibrinogen, hypertension, and self‐reported chest infection. Observations with a fractional probability of being eligible to the Winter Fuel Payment are dropped. CRP = C‐reactive protein

**Table 2:** The impact of the Winter Fuel Payment on predictors of infection

Effect of the Winter Fuel Payment on fibrinogen, C‐reactive protein, self‐reported chest infection, and hypertension				
	Fibrinogen	C‐reactive protein	Self‐reported infection	Hypertension
Causal effect of eligibility [95% confidence interval]	−058*** [−.080, −036]	−013 [−040, .015]	−.024* [−050, .001]	−018 [−045, .009]
Minimum detectable effect (at 80% power)	±0.028	±0.034	±0.032	±0.034
Unadjusted *p* value (adjusted for multiple testing)	0.000 (0.022)	0.418 (0.220)	0.058 (0.091)	0.207 (0.119)
Number of observations	3,974	4,517	4,569	6,295
Age window	55–65	55–65	55–65	55–65

*Note*. Standard Errors clustered by age of the oldest household member. The regression discontinuity designs have a linear specification in the age of the oldest member in the household. Additional covariates are type of household, gender, smoker status, alcohol consumption, body mass index, waist circumference, education, income, employment status, month of nurse visit, and survey‐wave dummies.

*** *p* < .01.

** *p* < .05.

* *p* < .1.

In Table 2, each column gives the estimate of the WFP effect on the health outcomes in our preferred specification with linear functions of the forcing variable, *f*(), covariates, and a sample age window (for the age of the oldest member in the household window) of 50 to 72 years. The point estimates suggest that the WFP improved the health of the elderly at age 61, reducing all our measures of illness. Effect sizes are 1 to 6 percentage points.

However, the WFP only has a statistically significant effect at 5% level on having a high concentration of fibrinogen. This finding is robust to the adjustment for multiple testing using the Romano–Wolf algorithm (see the third line in Table 2). The WPF decreases the probability of a fibrinogen value in excess of 4 g/L by 5.8 percentage points. As 12.5% of the elderly just below the cut‐off have a value of fibrinogen in excess of four, our estimate implies a 46% reduction in the incidence of this measure of illness at the age cut‐off. Data on the CRP are noisier, and the discontinuity effect at the cut‐off is not statistically significant at conventional levels. Nonetheless, the magnitude of the WFP effect (1.3 percentage points) is sizeable and implies a 24% reduction in the incidence of illness by this measure, again at the cut‐off. 14The fraction of elderly just below the age 61 cut‐off with CRP value larger than 10 is 5.5%.


In column 3, we report the effect on self‐reporting a chest infection in the last 3 weeks, and in column 4, we report the effect on the incidence of hypertension. The effects are about 2 percentage points, but neither is statistically significant at 5% level.

The effect on incidence of fibrinogen above the normal range is both the largest point estimate and the most precisely estimate effect. To get an idea of the power of our tests, we calculated, for each outcome, the minimum effect size we would have 80% power to detect. These are displayed in the second row of Table 2. Although we have quite large samples sizes, minimum detectable effects are quite large. This is partly because we are examining the incidence of extreme values, and partly because we only have clean identification of treatment effects at the eligibility cut‐off and must model the evolution of illness incidence on either side of the cut‐off.

To increase our confidence in the main result reported in Table 2, we conducted several robustness checks including: omitting covariates, varying the data window or the degree of the polynomial in the running variables, and omitted those households whose eligibility could only be determined probabilistically. Our main result of a significant effect on extreme values of fibrinogen and is robust to all of these variations. We also conducted falsification tests. We found no discontinuity in the incidence of extreme values of fibrinogen at placebo age cut‐offs. We also found no effect of WFP eligibility on the median value of fibrinogen—indicating that effect is just on the incidence of extreme values, indicative of acute illness. Finally, a potential concern is that even though no member of our sample qualifies for a state pension at the eligibility cut‐off, there could still be discrete changes in labour supply (that is, retirement) when a man reaches age 60 (see Beatty et al., 2014 for further discussion). We address this in our main estimates by including employment status in our additional covariates. Additionally, our balance tests include treating employment status as the outcome in an RDD regression analogous to those we estimate for health outcomes. We find no evidence of discrete change in employment status at the WFP eligibility cut‐off. Full details of our robustness checks, falsification tests, and balance tests are available in the Supporting Information.

Turning to subsample analysis, we follow Kling, Liebman, and Katz (2007) and create a poor health index and implement the RDD on this new measure. This both improves the statistical power of detecting effects of the same sign and reduces problems of multiple testing. The poor health index combines our indicators of cardiovascular and respiratory diseases in a single measure. We exclude self‐reports of chest infection and focus on our binary objective measures, serum‐fibrinogen in excess of four, serum‐CRP in excess of 10, and hypertension. 15Self‐reports of chest infection are the only measure that does not show a clear pattern in the data and is not reported in HSE. For each of these outcomes, we calculate the *z*‐scores subtracting the mean of the group of people just below the eligibility cut‐off and dividing by the standard deviation of the same group. The poor health index is the average of the three *z*‐scores, and a low value of the index is evidence of better health.

**Figure 5:**
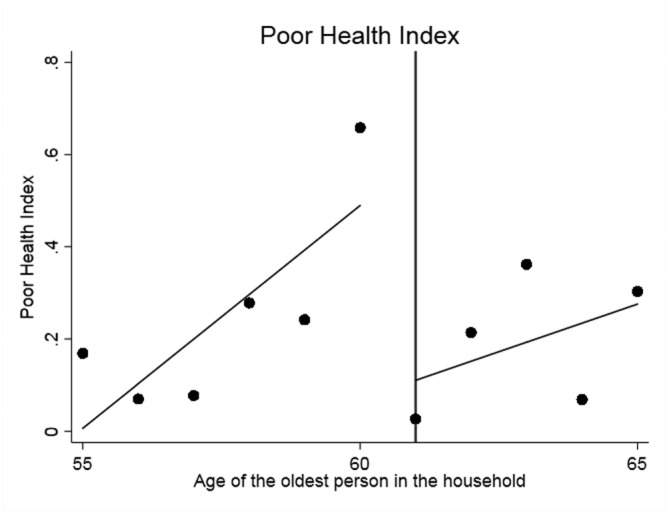
Effect of the Winter Fuel Payment on the poor health index. Plotted points represent the mean of the poor health index by year of age of the oldest person in the subject's household, with best fit regression lines to the left and right of the eligibility cut‐off

We first present the impact of the WFP on the poor health index graphically in Figure 5. As in the figures reported previously, a discontinuity at the eligibility cut‐off is evidence of an effect of the WFP on health. The data in Figure 5 are still noisy as in the analysis of the single objective health outcomes, but there is a visible drop in the poor health index around the eligibility cut‐off, indicating improved health.

In Table 3, we show the size of the effect of the WFP on the poor health index for different subsamples. An estimate for the full sample is presented in column 1 and shows a statistically significant decrease in the poor health index of 0.23 standard deviations. In the Supporting Information, we show that the reduction of the poor health index is in the range of 0.2 to 0.51 standard deviations and robustly statistically significant at 5% level when we change our RDD specification in the ways listed above.

**Table 3:** Impact of the Winter Fuel Payment on the poor health index in subgroups

All sample	Colder months only	Low‐income (first quartile)	Low education
−232*** [−384, −081]	−384* [−786, .017]	−044 [−422, .334]	−485*** [−818, −152]
*N* = 3481	*N* = 2351	*N* = 786	*N* = 980

*Note*. Standard Errors clustered by age of the oldest member level; 95% confidence interval. Linear specification in age of the oldest household member. Age window: 55–65. Colder months exclude June, July, August, and September. Low‐educated highest qualification reported: No qualification, NVQ Level 1, NVQ Level 2. Additional covariates are type of household, gender, smoker status, alcohol consumption, body mass index, waist circumference, education, income, employment status, month of nurse visit, and survey‐wave dummies.

*** *p* < .01.

** *p* < .05.

* *p* < .1.

In the second column of Table 3, we report the estimates on a sample, which excludes observations with a nurse visit in summer. The rational is that we do not expect any beneficial effect of the WFP on health in the summer months. Consistent with this expectation, we find a slightly larger reduction in the poor health index than in the full sample (a point estimate of 0.38 standard deviations that is statistically significant at 10% level). Next, we estimate the effect of the WFP on the poor health index for low‐income respondents (column 3). Beatty et al. (2014) show a particularly significant increase in the fuel expenditure for poorer households. These households should therefore experience larger health benefits. We find an effect magnitude of −0.04 standard deviations that is not precisely estimated, in part because of the reduction in the sample size. Finally, we consider the effect of WFP eligibility among low education respondents. Here, we find a very large and statistically significant effect of around 0.49 standard deviations. For older households, education may be a better indicator of economic resources than current income. Alternatively, low education households may be more susceptible to effect of the labelling.

## 4 DISCUSSION

We find evidence to suggest that raising the cut‐off age for WFP eligibility has had a negative effect on the health of individuals made ineligible. We find a robust and statistically significant effect for only one of the individual illness measures we consider, though point estimates for all the markers we consider point in this direction. Reductions of 1 to 6 percentage points (for fairly rare events) are large effect sizes. Using a poor health index that combines our illness markers, we find particularly large effects for low education individuals.

For healthcare providers, these results highlight the need to be sensitive to inadequate indoor heating as a potential winter health risk, perhaps particularly among low education individuals who fall just short of eligibility for the WFP.

For policy makers, our results suggest that the tightening of eligibility for the WFP that has come with increases to the female state pension age may not have come without health costs. Our RDD estimates indicate a causal effect of the WFP on the health of individuals living in households where the oldest member of the household is 61. This is precisely the age group that lost any health benefits of the WPF as the eligibility age was incrementally increased from 2010 (that is, the group that would have been eligible had the eligibility age not increased, but now is not). Our subsample analysis, finding particularly large effect for low education individuals, may point to means testing as a better way of rationing the programme. At a minimum, further research is needed to determine the loss of health benefits associated with potential future increases in the eligibility age.

## ETHICAL STATEMENT

This secondary data analysis did not require ethics review but the underlying surveys all obtained ethics approval. (Waves 2 and 4 of ELSA, 2001, 2003, 2004, 2006 HSE: MREC; HSE 2009: Oxford B Ethics committee; 2003 SHeS: MREC for Scotland; 2008, 2009 SHeS: MREC for Wales).

## ORIGINAL PUBLICATION

This paper is the original work of the authors, and no part of it has been submitted for publication to any other journal.

## CONFLICT OF INTEREST

Neither author has nor has had a financial relationship with any organisations that might have an interest in the submitted work; no other relationships could appear to have influenced the submitted work.

## Supporting information

Table A.1. Winter Fuel Payment eligibility.
Table B.1 Robustness checks. The impact of the Winter Fuel Payment on predictors of infection.
Table B.2 Falsification Tests: Effect of a “placebo” eligibility at age 55 and age 65, and effect on above median fibrinogen concentration.
Table B.3 Balance Tests: Effect on Employment Status.
Table C.1 The impact of the Winter Fuel Payment on the Poor Health Index.
