# Supplementary material for: The health benefits of a targeted cash transfer: The UK Winter Fuel Payment
Source: Health Econ. 2018 May 9;27(9):1354–65. doi: 10.1002/hec.3666 (PMC6099427; doi:10.1002/hec.3666)
Supplement: Supplementary file 1 — Table A.1. Winter Fuel Payment eligibility. Table B.1 Robustness checks. The impact of the Winter Fuel Payment on predictors of infection. Table B.2 Falsification Tests: Effect of a “placebo” eligibility at age 55 and age 65, and effect on above median fibrinogen concentration. Table B.3 Balance Tests: Effect on Employment Status. Table C.1 The impact of the Winter Fuel Payment on the Poor Health Index. [file HEC-27-1354-s001.docx]

1. **Eligibility for WFP**

Eligibility for the WFP is determined by the age of the oldest household member in the preceding September. Thus a respondent’s household will have received a WFP in the December prior to the nurse visit date only if the oldest member of the household was 60 in the September immediately before that December. All households with an oldest member aged 59 or less at the date of the nurse visit will not have received a WFP. All households with an oldest member aged 62 or more at the date of the nurse visit will have been eligible for at least one WFP, and, given the very high take-up this benefit, almost surely received it. For households with an oldest member aged 60 or 61, whether they have been eligible for a WFP will depend on both the date of the nurse visit and the birthday of the oldest member of the household. A complication is that, although the month of the nurse visit is known, ages are recorded in the data in years. That means, the WFP status of some households with an oldest member aged 60 or 61 can only be determined probabilistically. This is described in Table A.1.^[[1]](#footnote-1)^ In our identification strategy we deal with this in two ways. First, we define $D_{it}$ according to Table A.1, so that $D_{it}=0$ if $A_{it}<59$, $D_{it}=1$ if $A_{it}>61$ and $D_{it}\in[0,\frac{1}{12},\frac{2}{12},\ldots1]$ if $A_{it}\in60,61$. Second, as a robustness check, we re-estimate the model dropping all observations for which WFP cannot be discretely determined. Note that when these cases are dropped, $D_{it}=1[A_{it}>60]$ (exactly), where 1[.] is an indicator function.

**Table A.1, Winter Fuel Payment eligibility.**

| **Month of the nurse visit** | **Eligibility age** | **WFP eligibility Aged 60** | **WFP eligibility Aged 61** | **WFP eligibility Aged 62** |
| --- | --- | --- | --- | --- |
| **January** | 60 + 4 months | 8/12 | 1 | 1 |
| **February** | 60 + 5 months | 7/12 | 1 | 1 |
| **March** | 60 + 6 months | 6/12 | 1 | 1 |
| **April** | 60 + 7 months | 5/12 | 1 | 1 |
| **May** | 60 + 8 months | 4/12 | 1 | 1 |
| **June** | 60 + 9 months | 3/12 | 1 | 1 |
| **July** | 60 + 10 months | 2/12 | 1 | 1 |
| **August** | 60 + 11 months | 1/12 | 1 | 1 |
| **September** | 61 | 0 | 1 | 1 |
| **October** | 61 + 1 months | 0 | 11/12 | 1 |
| **November** | 61 + 2 months | 0 | 10/12 | 1 |
| **December** | 61 + 3 months | 0 | 9/12 | 1 |

1. **Individual Measures: Robustness Checks, Falsification Test, Balance Tests**

In Table B.1 we explore the robustness of our results by varying our RDD specification in 5 ways. We first implement a quadratic polynomial for *f( )*, the function of the forcing variable relative to the age cut-off. We then re-estimate the models without including covariates in our specification. We further investigate whether our findings are sensitive to a change in the choice of the sample age window (either wider or narrower).

**Table B.1 Robustness checks. The impact of the Winter Fuel Payment on predictors of infection.**

| **Effect of the WFP on Fibrinogen, C-reactive protein, Self-reported Chest Infection and Hypertension** | | | | |
| --- | --- | --- | --- | --- |
|  | **Fibrinogen** | **C-reactive protein** | **Self-reported infection** | **Hypertension** |
| **Quadratic specification in age of oldest household member**  **[95% Confidence Interval]** | -.084**  [-.164; -.005] | -.032  [-.079; .016] | .001  [-.025; .027] | -.089***  [-.133; -.045] |
| **No additional covariates**  **[95% Confidence Interval]** | -.048***  [-.071; -.026] | -.010  [-.039; .020] | -.027*  [-.058; .005] | -.014  [-.040; .011] |
| **Narrower Age Window: 57-63**  **[95% Confidence Interval]** | -.093***  [-.139; -.047] | -.036**  [-.062; -.009] | -.016**  [-.031; -.000] | -.051**  [-.090; -.012] |
| **Wider Age Window: 50-70**  **[95% Confidence Interval]** | -.045***  [-.069; -.022] | -.015  [-.037; .006] | .016  [-.009; .041] | -.020  [-.051; .010] |
| **Dropping observations whose eligibility cannot be discretely determined**  **[95% Confidence Interval]** | -.064***  [-.094; -.034] | -.012  [-.043; .019] | -.015  [-.045; .016] | -.032  [-.073; .008] |

Standard Errors clustered by age of the oldest member level. The age window for the specifications in rows (1), (2) and (5) is 55-65. Additional covariates are type of household, gender, smoker status, alcohol consumption Body Mass Index, waist circumference, education, income, employment status, month of nurse visit, and survey-wave dummies.

*** p<0.01 **p<0.05 *p<0.1

Finally we drop the observations with the oldest member of the household aged 60 or 61 for whom we cannot determine whether they received the WFP or not exactly. We find that our estimates of the discontinuity effect for the fibrinogen are robust to any of these changes in the RDD specification. The coefficient of the WFP effect is always statistically significant at 5 % level and the effect size lies between -0.045 percentage points and -0.093 percentage points.^[[2]](#footnote-2)^ This implies a reduction of 36 % to 74 % in the incidence of a high serum concentration of fibrinogen at the age cut-off. For the other measures of illness we find a negative coefficient in all the specifications indicating an improvement in health with WFP eligibility. However, the estimates are variable and rarely statistically significant at conventional levels.

**Table B.2 Falsification Tests: Effect of a “placebo” eligibility at age 55 and age 65, and effect on above median fibrinogen concentration.**

|  | **Effect of WFP on Fibrinogen** |
| --- | --- |
| **Cut-off age 55**  **[95% Confidence Interval]** | -.024  [-.070; .022] |
| **Age Window** | 50-60 |
| **Cut-off age 65**  **[95% Confidence Interval]** | .034  [-.030; .098] |
| **Age Window** | 60-70 |
| **Prob(Fibrinogen≥3.1)**  **[95% Confidence Interval]** | .009  [-.044; .063] |
| **Age Window** | 55-65 |

Standard Errors clustered by age of the oldest member level. Additional covariates are type of household, gender, smoker status, alcohol consumption Body Mass Index, waist circumference, education, income, employment status, month of nurse visit, and survey-wave dummies.

*** p<0.01 **p<0.05 *p<0.1

We now provide some further checks on our main finding of a WFP effect on the incidence of high concentrations of fibrinogen. In Table B.2 we present falsification tests for an effect at age cut-offs of 55 and 65. As these are not the eligibility cut-off, we should find no effect at these ages. As a further falsification test we check for an effect on the incidence of fibrinogen concentrations above the sample median. The idea here is that having an above-median concentration of fibrinogen is not a marker of disease. If we are measuring a reduction in disease incidence, that effect should be observed only in the upper tail of the distribution (as in our main estimates) and not around the median.^[[3]](#footnote-3)^ As Table B.2 illustrates, we do not find any evidence of an effect across these specifications. This increases our confidence in the baseline results.

We also considered what our estimated effects for the incidence of fibrinogen concentration in excess of 4g/l imply for levels fibrinogen in the upper tail of the distribution. To do this we estimated a quantile regression version of the RDD. Note that our base specification studies the probability that measured serum Fibrinogen exceeds a specified cut-off, and how this probability differs with WFP eligibility holding the cut-off constant at *k*.:

$$P_{it}\left( WFP_{it} \right)=Prob(H_{it}^{Fib}>k|WFP_{it})$$

A quantile regression inverts this relationship, holding the probability constant (at the chosen quantile, *1 - P*) and asking, essentially, how the cut-off varies with WFP eligibility.

$$k_{it}=F_{1-P}(H_{It}^{Fib}|WFP_{it})$$

In our sample $prob\left( H_{It}^{Fib}=1 \right)\approx12\%$ and the RDD estimates show that this falls by 6.1 percentage points with WFP eligibility. In this robustness check we consider how the 85^th^ and 90^th^ conditional quantiles of $H_{It}^{Fib}$ vary with WFP eligibility (corresponding to *P = 0.15* and *P = 0.1*). We find in both cases a drop in Fibrinogen of about 0.11 g/l. However the effects are less precisely estimated than the probability models.^[[4]](#footnote-4)^

**Table B.3 Balance Tests: Effect on Employment Status.**

| **Pooled Data** | **Men** | **Women** |
| --- | --- | --- |
| -.001  [-.095; .092] | 0.029  [-.079; .136] | -.047  [-.129; .036] |
| N= 7317 | N= 4312 | N= 3005 |
| Age Window:  55-65 | Age Window:  55-65 | Age Window:  55-65 |

Standard Errors clustered by age of the oldest member level. 95% Confidence Interval. Additional covariates are type of household, gender, smoker status, alcohol consumption Body Mass Index, waist circumference, education, income, month of nurse visit, and survey-wave dummies.

*** p<0.01 **p<0.05 *p<0.1

We also conducted balance tests (around the eligibility cut off) for all of the covariates in listed in the main text. One deserves particular mention. A possible concern is that changes in employment status at the age of 60 can confound our results. Our sample excludes single women and couples in which the woman is older, so that WFP eligibility is not coincident with a member of the household qualifying for the state pension. Nevertheless, 60 could be a focal retirement age for men. Our main estimates control for employment status, but we also conducted balancing tests with a similar RDD specification as our main results, but with employment status as our dependent variable. For our sample of single men and couples in which the men is the oldest there is no evidence of a discontinuity in employment at age 60. Nor did we find discontinuities in any of the other covariates.

1. **Poor Health Index: Robustness Checks**

In Table C.1 we compare our findings on the Poor Health Index by varying our RDD specification in the same way we conducted robustness checks on single health outcomes in Appendix B. In Column 2 we implement a quadratic polynomial for the function of the forcing variable *f( )*, in Column 3 and 4 we change the choice of the sample age window and in Column 5 we drop the observations whose WFP eligibility cannot be determined exactly.

We find that the WFP reduces the Poor Health Index in the range of 0.2 to 0.51 standard deviations. All the point estimates in Table C.1 are statistically significant at 5 % level.

**Table C.1 The impact of the Winter Fuel Payment on the Poor Health Index.**

| **Linear Specification in age of oldest household member** | **Quadratic specification in age of oldest household member** | **Narrower Age Window** | **Wider Age Window** | **Dropping observations whose eligibility cannot be discretely determined** |
| --- | --- | --- | --- | --- |
| -.232***  [-.384; -.081] | -.512**  [-.876; -.149] | -.475***  [-.682; -.268] | -.200**  [-.377; -.022] | -.302**  [-.551; -.054] |
| N= 3481 | N= 3481 | N= 2343 | N= 5709 | N= 3142 |
| Age Window:  55-65 | Age Window:  55-65 | Age Window: 57-63 | Age Window: 50-70 | Age Window:  55-65 |

Standard Errors clustered by age of the oldest member level. 95% Confidence Interval. Additional covariates are type of household, gender, smoker status, alcohol consumption Body Mass Index, waist circumference, education, income, employment status, month of nurse visit, and survey-wave dummies.

*** p<0.01 **p<0.05 *p<0.1

1. For example, if an individual has the nurse visit in January, their household will have received a WFP in December (one month before), as long as the oldest member is more than 60 years and 4 months old, so that they were 60 in the preceding September. If the oldest member of the household reports age 60 and was born in August, the household will have been eligible for a WFP in December. However, if the oldest member of the household reports age 60 and was born in October, the household will not have been eligible for a WFP in December. Of those oldest members of a household aged 60 in years at a given nurse visit, 2/3 will be older than 60 years and 4months, and 1/3 will be 60 years four months or less. [↑](#footnote-ref-1)
2. All estimates are statistically significant at 5% level after adjusting for multiple testing except for the specification with a quadratic function of the forcing variable, where p=0.074. [↑](#footnote-ref-2)
3. The median fibrinogen in the neighbourhood of the age cut-off is 3.1 (see Table 1). [↑](#footnote-ref-3)
4. Inference for quantile regression is not straight forward. Asymptotic standard errors are not regarded as reliable and we employ a bootstrap procedure. Full details and results are available from the authors on request. [↑](#footnote-ref-4)
